# Comparability, acceptability and longitudinal adherence with digital emPHasis-10 in pulmonary arterial hypertension

**DOI:** 10.1183/13993003.00198-2025

**Published:** 2025-06-19

**Authors:** Joseph Newman, Frances Varian, Felicity Hitchcock, Rebecca Burney, Gregg Harry Rawlings, John Harrington, Ze Ming Goh, Jenna Ablott, David G. Kiely, Iain Armstrong, A.A. Roger Thompson, Jill Carlton, Elin Haf Davies, National Cohort Study of Pulmonary Hypertension Collaboration, UniPHy Clinical Trials Network, Alexander Rothman, Mark Toshner

**Affiliations:** 1 Victor Phillip Dahdaleh Heart and Lung Research Institute, University of Cambridge, Cambridge, UK; 2 Royal Papworth Hospital, Cambridge, UK; 3 Division of Clinical Medicine, School of Medicine and Population Health, University of Sheffield, Sheffield, UK; 4 Sheffield Pulmonary Vascular Disease Unit, Royal Hallamshire Hospital, Sheffield Teaching Hospitals NHS Foundation Trust, Sheffield, UK; 5 Clinical and Applied Psychology Unit, University of Sheffield, Sheffield, UK; 6 Pulmonary Hypertension Association United Kingdom, Sheffield, UK; 7 Sheffield Centre for Health and Related Research (SCHARR), University of Sheffield, Sheffield, UK; 8 Aparito Ltd, Wrexham, UK; 9 J. Newman and F. Varian are joint first authors; 10 A. Rothman and M. Toshner are joint last authors

## Abstract

Pulmonary hypertension (PH) affects 1% of the global population and significantly impacts health-related quality of life (HRQoL) [1, 2]. Patient-reported outcome measures (PROMs) are standardised tools used in clinical practice and research to assess health outcomes from the patient's perspective. Routine measurement of HRQoL is supported by clinical guidelines, which recommend disease-specific PROMs [1]. EmPHasis-10 is a widely used 10-item PROM developed for patients in any World Health Organization (WHO) PH group [2, 3]. Available in numerous languages, it has strengths in both its psychometric properties and feasibility [2, 4]. However, it is currently only available in a paper-based format.

## Shareable abstract

**Digital emPHasis-10 is an app-based patient-reported outcome measure for trial or clinical use by patients with pulmonary hypertension. This letter demonstrates this novel tool's validity, longer-term adherence and high acceptability scores from patients.** https://bit.ly/4lZiXkE

*To the Editor:*

Pulmonary hypertension (PH) affects 1% of the global population and significantly impacts health-related quality of life (HRQoL) [1, 2]. Patient-reported outcome measures (PROMs) are standardised tools used in clinical practice and research to assess health outcomes from the patient's perspective. Routine measurement of HRQoL is supported by clinical guidelines, which recommend disease-specific PROMs [1]. EmPHasis-10 is a widely used 10-item PROM developed for patients in any World Health Organization (WHO) PH group [2, 3]. Available in numerous languages, it has strengths in both its psychometric properties and feasibility [2, 4]. However, it is currently only available in a paper-based format.

Electronic PROMs (ePROMs), such as those delivered on smartphone applications (apps), are recommended by international stakeholders and regulatory bodies [5–8]. Advantages include improved data integrity and accuracy, facilitation and tracking of “skip” patterns, high acceptability, better compliance, increased power leading to smaller sample sizes, and easier processing [9]. The capacity for patients to use their own devices and the ubiquity of smartphones could reduce trial delivery costs while maintaining equitable access. Offering a choice of PROM formats is expected to enhance inclusion and engagement [8, 10, 11].

International guidelines recommend evaluation of alternative PROM formats to ensure that measurement properties do not change, and only recommend full psychometric validation where certain criteria are met [8, 9]. We report the first study evaluating digital and paper emPHasis-10 equivalence, longitudinal adherence and acceptability from the patients’ perspective.

As part of a UK multicentre prospective observational study, adult patients with pulmonary arterial hypertension (PAH) consented to participate in Cohort-Digital (IRAS 123349, REC 13/EE/0203) and/or Feasibility of Novel Clinical Trial Infrastructure, Design and Technology for Early Phase Studies in Pulmonary Hypertension (FIT-PH; NCT04078243, REC 19/YH/0354). Participants completed the paper-based emPHasis-10 and the digital format *via* the Atom5 app (iOS or Android) as a “bring your own device” study [12]. Participants received fortnightly pre-programmed push notifications (alerts) for a 26-week period asking them to remotely complete the digital emPHasis-10. Passive compliance was audited, with no additional active adherence interventions deployed. Patients could contact the study team for technical assistance if required, and although not audited systematically, this was rare.

A predominantly prevalent and stable PAH population was prioritised. International PROM development guidelines recommend a minimum sample size of 50 for evaluation of measurement error [13]. Stability was determined by a patient-reported neutral score (−1, 0 or +1) on a digital subjective global anchor rating scale (−3 to +3) asking “*with respect to your pulmonary hypertension, how would you describe yourself NOW compared to when you last completed this questionnaire?*” The UTAUT (Unified Theory of Acceptance and Use of Technology) underpinned a digital survey to evaluate themes of usability and acceptability [14].

Digital emPHasis-10 was developed and tested with focus groups of patients with PH in collaboration with Pulmonary Hypertension Association UK. Following international recommendations, full psychometric evaluation was not required as the format change from paper to digital was deemed mild to moderate [9]. These “non-substantive” formatting differences included: 1) change in instructions from “placing a tick” (boxes) to “selecting the number” (Likert scale), 2) change from 10 items on a single sheet to one item per screen, 3) no total score immediately visible on the digital version and 4) automated date and time stamping of digital completion.

51 patients were enrolled: median age was 53 years (interquartile range (IQR) 41–62 years), 71% were female, and 81% were white. Most (41 out of 51; 80%) had a diagnosis of idiopathic PAH and the median time since diagnosis was 5 years (IQR 1–11.5 years). 82% of patients had a low/intermediate-low COMPERA 2.0 risk score with WHO functional class I/II/III/IV 7/41/50/2%, respectively. The cohort was geographically diverse, with patients enrolled from across the UK.

57 pairs of digital/paper PROMs (from multiple clinic visits) were available for evaluation from stable patients. Median time between digital and paper completion was +1 day (IQR 0 to 5) with a range of up to 31 days between formats where patients reported no significant change in their HRQoL as evaluated using the patient-reported anchor score. Paired samples from two participants were excluded after reporting they completed the digital format incorrectly, accidentally inverting the scales. Samples where patients reported a change in HRQoL on the global anchor scale were not included for equivalence comparison.

Mean scores were equal at 20 out of 50 (±14) with standard error of measurement (sem) of 2. Scores were consistent between paper and digital formats (Spearman's r=0.98, p<0.0001, Cronbach's alpha 0.99). Bland–Altman analysis (n=57) showed a bias (systematic error) of 0.1 (sd 2.3) with 95% limits of agreement from −4.7 to 4.4 (figure 1a). All random variation fell below the thresholds estimated to be the minimal clinically important difference (MCID) (figure 1a) [15, 16].

**FIGURE 1:**
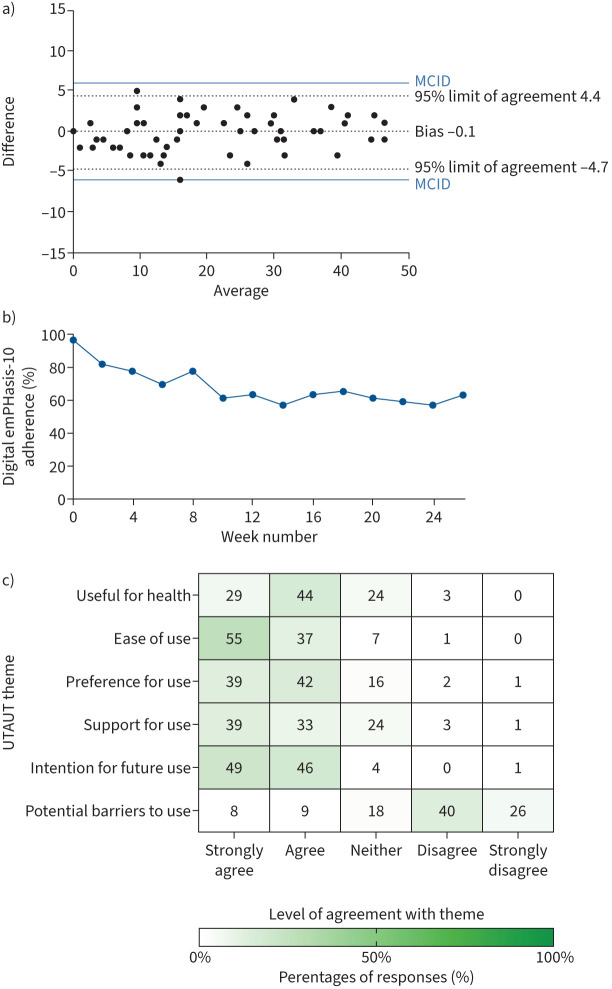
a) Bland–Altman plot showing the level of agreement between digital and paper-based emPHasis-10. For reference, 50 is the maximum possible emPHasis-10 score. b) Plot of patient adherence with digital emPHasis-10 each fortnight for 6 months. c) Heatmap showing the percentage of patients who agree with each of the key usability/acceptability themes from the modified UTAUT (Unified Theory of Acceptance and Use of Technology) survey. MCID: minimal clinically important difference.

The overall adherence (Cohort-Digital n=49) to completing fortnightly ePROMs was a median of 79% (IQR 29–100%) and 29% (14 out of 49) of patients achieved 100% compliance. This included one participant who withdrew from the study before accessing the app. There was a steady drop-off in completion during the first 6 weeks of the study before reaching a plateau of 61% by 26 weeks (figure 1b).

Patient-reported acceptability of the app-based emPHasis-10 was high (figure 1c), consistent with other studies showing preferences for ePROMs [17]. Most participants reported finding it useful, usable, preferable and would use it again in clinical practice or research if available, without major barriers to adoption.

The primary finding from this study is that patients responded consistently between paper and digital formats of emPHasis-10. A further strength is the consistency of PROM scores with patient-reported stability, highlighting the value of anchor-based methodology. Based on the strong association, consistency and acceptability metrics, we suggest that paper or digital formats can be selected in accordance with patient preference, trial design or clinical setting.

The small sem (score of 2) of digital emPHasis-10 during a period of self-reported stability suggests that a threshold of >2 could be significant. Low scoring variability suggests that emPHasis-10 may be sensitive to changes below the registry-estimated MCIDs of 6 to 8 [15, 16]. Evaluation of responsiveness of digital emPHasis-10 is underway through ongoing therapeutic trials [18, 19].

Most patients regularly used the ePROM. The observed drop-off over 6 months is a recognised phenomenon [17]. The modest initial disengagement could be mitigated by making the tool seemingly more interactive or useful, such as diarising ePROM scores over time – a function we have subsequently co-developed with patients. This tracker function also aims to reassure patients that data is not lost, a concern expressed on the UTAUT survey. An “investigator in the loop” design, rather than solely notification-driven reminders, is recommended to maximise completion rates, address technical issues and prevent drop-off, as supported by our usability data and the literature [20]. Strategies to optimise longitudinal adherence are ongoing through the Cohort-Digital randomised study.

In conclusion, this is the first equivalence evaluation of a digital format of emPHasis-10. Established using a patient-reported anchor question, this methodology follows international recommendations and strengthens the field in HRQoL outcome measurement, with broad applicability. This ePROM is highly acceptable to patients with PAH and has reasonable adherence longer-term. The digital format will allow for more frequent, convenient and remote collection of meaningful HRQoL data in both clinical practice and trials.

## Shareable PDF

10.1183/13993003.00198-2025.Shareable1 This PDF extract can be shared freely online.
Shareable PDF

ERJ-00198-2025.Shareable
