# Supplementary material for: Comparability, acceptability and longitudinal adherence with digital emPHasis-10 in pulmonary arterial hypertension
Source: Eur Respir J. 2025 Jun 19;65(6):2500198. doi: 10.1183/13993003.00198-2025 (PMC12177334; doi:10.1183/13993003.00198-2025)
Supplement: Supplementary file 1 [file ERJ-00198-2025.Shareable.pdf]

SHAREABLE PDF

Comparability, acceptability and longitudinal adherence with digital emPHasis-10 in pulmonary arterial hypertension

Joseph Newman [1,2,9], Frances Varian [3,4,9], Felicity Hitchcock [3,4], Rebecca Burney [3,4], Gregg Harry Rawlings [5], John Harrington [4], Ze Ming Goh [3,4], Jenna Ablott [4], David G. Kiely [3,4], Iain Armstrong [3,4,6], A.A. Roger Thompson [3,4], Jill Carlton [7], Elin Haf Davies [8], National Cohort Study of Pulmonary Hypertension Collaboration, UniPHy Clinical Trials Network, Alexander Rothman [3,4,10] and Mark Toshner [1,2,10]

[1]Victor Phillip Dahdaleh Heart and Lung Research Institute, University of Cambridge, Cambridge, UK. [2]Royal Papworth Hospital, Cambridge, UK. [3]Division of Clinical Medicine, School of Medicine and Population Health, University of Sheffield, Sheffield, UK. [4]Sheffield Pulmonary Vascular Disease Unit, Royal Hallamshire Hospital, Sheffield Teaching Hospitals NHS Foundation Trust, Sheffield, UK. [5]Clinical and Applied Psychology Unit, University of Sheffield, Sheffield, UK. [6]Pulmonary Hypertension Association United Kingdom, Sheffield, UK. [7]Sheffield Centre for Health and Related Research (SCHARR), University of Sheffield, Sheffield, UK. [8]Aparito Ltd, Wrexham, UK. [9]J. Newman and F. Varian are joint first authors. [10]A. Rothman and M. Toshner are joint last authors.

Corresponding author: Joseph Newman (joseph.newman@nhs.net)

Shareable abstract (@ERSpublications)
Digital emPHasis-10 is an app-based patient-reported outcome measure for trial or clinical use by patients with pulmonary hypertension. This letter demonstrates this novel tool's validity, longer-term adherence and high acceptability scores from patients. https://bit.ly/4lZiXkE

Cite this article as: Newman J, Varian F, Hitchcock F, *et al*. Comparability, acceptability and longitudinal adherence with digital emPHasis-10 in pulmonary arterial hypertension. *Eur Respir J* 2025; 65: 2500198 [DOI: 10.1183/13993003.00198-2025].

This PDF extract can be shared freely online.

Check for updates

Copyright ©The authors 2025.

This version is distributed under the terms of the Creative Commons Attribution Licence 4.0.

Received: 29 Jan 2025
Accepted: 26 April 2025

To the Editor:

Pulmonary hypertension (PH) affects 1% of the global population and significantly impacts health-related quality of life (HRQoL) [1, 2]. Patient-reported outcome measures (PROMs) are standardised tools used in clinical practice and research to assess health outcomes from the patient's perspective. Routine measurement of HRQoL is supported by clinical guidelines, which recommend disease-specific PROMs [1]. EmPHasis-10 is a widely used 10-item PROM developed for patients in any World Health Organization (WHO) PH group [2, 3]. Available in numerous languages, it has strengths in both its psychometric properties and feasibility [2, 4]. However, it is currently only available in a paper-based format.
